# Bronchiectasis in Primary Antibody Deficiencies: A Multidisciplinary Approach

**DOI:** 10.3389/fimmu.2020.00522

**Published:** 2020-03-31

**Authors:** Luke A. Wall, Elizabeth L. Wisner, Kevin S. Gipson, Ricardo U. Sorensen

**Affiliations:** ^1^Division of Allergy Immunology, Department of Pediatrics, Louisiana State University Health Sciences Center New Orleans, New Orleans, LA, United States; ^2^Children's Hospital of New Orleans, New Orleans, LA, United States; ^3^Division of Pulmonology and Sleep Medicine, Department of Pediatrics, Massachusetts General Hospital, Harvard Medical School, Boston, MA, United States

**Keywords:** bronchiectasis, antibody deficiencies, primary immunodeficencies (PID), immunoglobulin replacement therapy (IgRT), pulmonary therapy, non-infectious complications

## Abstract

Bronchiectasis, the presence of bronchial wall thickening with airway dilatation, is a particularly challenging complication of primary antibody deficiencies. While susceptibility to infections may be the primary factor leading to the development of bronchiectasis in these patients, the condition may develop in the absence of known infections. Once bronchiectasis is present, the lungs are subject to a progressive cycle involving both infectious and non-infectious factors. If bronchiectasis is not identified or not managed appropriately, the cycle proceeds unchecked and yields advanced and permanent lung damage. Severe symptoms may limit exercise tolerance, require frequent hospitalizations, profoundly impair quality of life (QOL), and lead to early death. This review article focuses on the appropriate identification and management of bronchiectasis in patients with primary antibody deficiencies. The underlying immune deficiency and the bronchiectasis need to be treated from combined immunology and pulmonary perspectives, reflected in this review by experts from both fields. An aggressive multidisciplinary approach may reduce exacerbations and slow the progression of permanent lung damage.

## Background

Primary antibody deficiencies are the most common inherited form of primary immunodeficiency (1). Disorders within this group, all of which involve impaired function of the B cell compartment, encompass a wide and rapidly growing array of underlying genetic causes. An intrinsic B cell defect may be the primary cause of the disorder (predominantly antibody deficiencies), or B cells may be unable to function optimally secondarily to numerous other forms of immune defects. Patients suffer from chronic and recurrent bacterial sinopulmonary infections with common respiratory pathogens such as non-typeable *Haemophilus influenzae* (NTHi) and *Streptococcus pneumoniae* (2). Infections with atypical bacteria (*Mycoplasma* and *Ureaplasma* species) as well as increased frequency and severity of common viral respiratory infections may occur (2). Patients should undergo laboratory workup to evaluate for a potential antibody deficiency when they experience sinopulmonary infections with unusual frequency, duration, or severity (3). The most profound example of a predominantly antibody deficiency is agammaglobulinemia, which has undetectable B cells (4). While X-linked agammaglobulinemia (XLA), due to absent Bruton's tyrosine kinase (BTK) was initially recognized to cause arrested B cell development, there are now at least 12 known molecular defects which lead to agammaglobulinemia (4). The most common clinically relevant predominantly antibody deficiency is common variable immune deficiency (CVID). CVID can be defined as the following: Low IgG and at least one other low isotype (IgA or IgM) with poor response to vaccines (5). CVID is not diagnosed in infants and toddlers, as young patients may have a transient antibody defect. Much debate has centered on the definition and diagnostic criteria of CVID, stemming primarily from the rapidly increasing knowledge on numerous underlying molecular causes of a similar immunologic phenotype. The most recent International Union of Immunological Societies (IUIS) expert committee report lists 20 molecular defects which have been documented to cause an antibody deficiency consistent with CVID (4). If a genetic cause for the antibody deficiency is identified, the patient is diagnosed with the specific genetic immune defect, not CVID (5). While autoimmunity and immune dysregulation may be seen in many forms of immune deficiency, these problems are particularly common in patients with CVID (25–30% of patients) (2). Up to 94% of patients with CVID have some degree of detectable lung abnormalities by chest computed tomography (CT) (6). The diagnosis of bronchiectasis is made in ~23% of patients with CVID, according to the European Society for Immunodeficiencies Registry, with a large variation in prevalence (0–66%) between centers (7). Because CVID accounts for up to one-third of all primary immunodeficiencies (8), much of the discussion throughout this manuscript focuses on this immune deficiency. It is essential, however, to realize that any clinically relevant antibody deficiency may lead to bronchiectasis. The IUIS regularly updates an extensive classification on antibody deficiencies which serves as an excellent resource (4).

**Figure 1:**
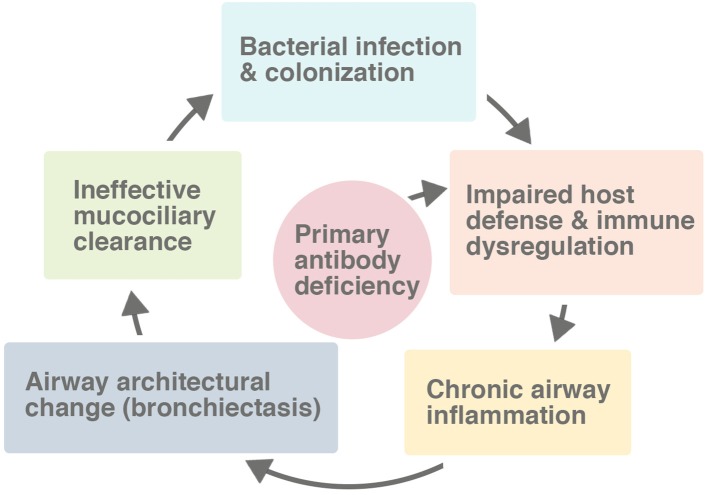
The vicious cycle leading to bronchiectasis in the setting of a primary antibody deficiency. While the host defect of the immune system impairs the response to pulmonary infections, the defect also directly causes immune dysregulation and a chronic inflammatory state in many patients. Many factors, including secondarily impaired mucociliary clearance, contribute to the process of progressive lung damage.

Bronchiectasis is a disorder of airway architecture associated with chronic inflammation, manifesting usually as irreversible dilatation of the bronchi (9). The bronchial dilatation causes difficulty clearing bacteria and mucus from the airway, contributing to persistent infection and inflammation. Airway damage may proceed in a vicious inflammatory cycle (Figure 1). Patients with antibody deficiencies are at high risk for bronchiectasis not only because they cannot defend the lungs effectively against infections, but also because they may have a dysregulated inflammatory response.

The lens through which bronchiectasis is viewed has changed over time. Laënnec first described bronchiectasis in 1819 (10), only 3 years after he invented the stethoscope. At that time, antibiotics were not available and severe chest infections such as tuberculosis were common. Such chest infections often resulted in bronchiectasis, if the patient survived. In the modern antibiotic era, bronchiectasis occurs almost exclusively in the setting of an underlying host defect. Primary immunodeficiency accounts for 12–34% of non-cystic fibrosis (CF) bronchiectasis (11). While up to half of non-CF bronchiectasis cases remain idiopathic, the low incidence of bronchiectasis in developed countries suggests that such cases have a yet to be identified infection or an underlying defect in mechanisms involving the immune system, local defenses, or mucociliary clearance. Bronchiectasis is the result of multiple pathophysiological processes which occur in patients with a diverse list of underlying disorders. The heterogeneous nature of this disorder has led some to argue that bronchiectasis is among the most complex and challenging disorders in respiratory medicine (12).

Although two centuries have passed since bronchiectasis was first described, there remains much to be learned. The pathophysiologic processes leading to bronchiectasis remain poorly understood, and patients may often experience significant delays in diagnosis. In children, the early signs of bronchiectasis are often misdiagnosed as asthma (13). Early, optimal treatment, arrest of progression, and, ultimately, reversal of bronchiectasis, are goals which have not been widely met (14). Specifically, the clinical approach to bronchiectasis in the setting of antibody deficiencies lacks sufficient studies and clinical guidelines. The goal of this review is to combine and summarize currently available evidence with expert opinion from both the pulmonology and immunology fields regarding the comprehensive, multifaceted approach to bronchiectasis diagnosis and treatment in patients with antibody deficiencies.

## The Impact: Morbidity, Mortality, and Quality of Life

For adult patients with bronchiectasis, age-adjusted mortality is more than twice that of the general population (15). Predictors of mortality include old age, low forced expiratory volume in one second (FEV_1_), low body mass index (BMI), history of hospitalizations, and frequent exacerbations (12). Progressive airway damage can lead to respiratory failure and death (16). Persistent daily respiratory symptoms, fatigue, frequent and prolonged hospitalizations, and the mostly incurable nature of the disorder may negatively impact patient quality of life (QOL) and places significant stress on the patient and caregivers (12).

## Pathophysiology: The Immune System, Inflammation, and the Lung

In patients with antibody deficiencies, the lung is often considered the organ most susceptible to damaging infections, as it presents a vast mucosal surface to the environment. The surface area of the adult human lung is approximately equal to one-half of a tennis court and is exposed to 10,000 L of environmental air every day (17). Considering that the lining of the lung is composed of a membrane so thin that it readily allows gas exchange, it is not surprising that patients with antibody defects may fail to fully defend the lung from infections. Infections often launch the vicious cycle of progressive lung damage which culminates into bronchiectasis (Figure 1). Globally, severe lower respiratory tract infections early in life are the leading cause of bronchiectasis, with measles and tuberculosis accounting for 25% of post-infectious pediatric bronchiectasis (18).

**Figure 2:**
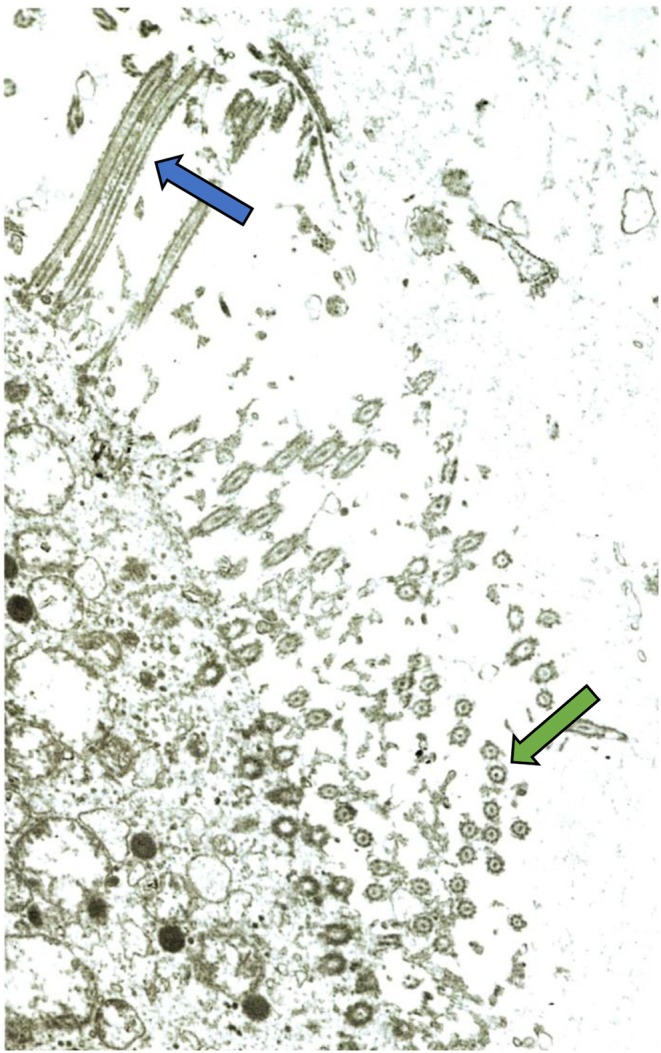
Electron micrograph of bronchial mucosa from a child with chronic chest symptoms. Despite normal intrinsic ciliary structure, a general paucity of cilia is demonstrated. This is a common finding on ciliary biopsy in the setting of recurrent respiratory infections. Green arrow indicates cilia seen in cross-section. Blue arrow indicates cilia seen lengthwise. Image courtesy Matthew Stark, MD, Pathologist, Children's Hospital New Orleans.

Mucociliary function may become secondarily impaired following the prolonged and recurrent infections experienced by patients with antibody deficiencies (12) (Figure 2). It is likely that weakened mucociliary function and biofilm formation play a significant role in driving chronic inflammation (12). Both local and systemic inflammation is increased in the setting of bronchiectasis and may persist even in the absence of infection (19). Within this inflammatory response, phagocytes and eosinophils have been identified as contributors to ongoing damage. Phagocyte recruitment is promoted by epithelial cells which release pro-inflammatory cytokines (20). Neutrophils and eosinophils contribute to airway inflammation and damage directly via degranulation (21). Although patients often have an increased number of airway macrophages, the ability of these cells to clear apoptotic neutrophils from the airway, as well as phagocytosis of NTHi, is impaired (22). Exhaled hydrogen peroxide (H_2_O_2_) has been used as a marker for activated neutrophils (23). Increased levels of H_2_O_2_ correlate with increased neutrophil burden, and worsened disease severity and lung function (23). Neutrophil elastase (NE), a pro-inflammatory serine protease released from azurophilic granules, may also be used as a marker of disease severity (24). NE slows ciliary beat frequency and increases mucus secretion, further contributing to the progression of disease (24). In the normal lung, NE is inhibited by anti-proteases such as alpha-1-antitrypsin (24). In bronchiectasis, release of NE overwhelms the anti-protease defense, leading to detectable levels of NE proteolytic activity in sputum (24). Linking pathogenesis with targeted therapies, NE is the focus of current trials for specific therapeutic agents in the treatment of bronchiectasis (25).

Bronchiectasis does not develop in all patients following lower respiratory tract infections, highlighting the essential concept that bronchiectasis encompasses many structural, functional, immunologic, and inflammatory processes. Interestingly, the neonatal Fc receptor (FcRn) may play a role. While this receptor is responsible for transplacental transfer of IgG from mother to fetus during pregnancy, it also plays a crucial role in IgG homeostasis throughout life (26). It is expressed on epithelial cells as well as vascular endothelial cells throughout the body (26). This transcytosis receptor maintains IgG in the circulation by facilitating the recycling of IgG, and is an important sensor of luminal infections (26, 27). In patients with CVID and bronchiectasis, lower FcRn mRNA expression was found to correlate with increased severity of bronchiectasis and a higher rate of IgG decline following intravenous immunoglobulin (IVIG) infusion (27). Studies of bronchiectasis in general, summarized below, have demonstrated that a disordered inflammatory response is an important variable, as is an impaired T cell response to pathogens. These are problems which are relatively common in some forms of antibody deficiencies such as CVID. A large observational study of CVID patients, for instance, demonstrated that 20% of CVID patients had low circulating CD4+ T cells, 40% had subnormal T cell proliferation response to one or more mitogens, and 22% demonstrated clinical autoimmune disease (28).

Cell-mediated immune response to NTHi, the most common respiratory pathogen in patients with bronchiectasis, has been found to be impaired in children with chronic suppurative lung disease (CSLD), which is considered a prelude to bronchiectasis. NTHi is unique in that it produces human IgA proteases which may contribute to the invasion of respiratory epithelium (18). It is also capable of intracellular survival in host macrophages and respiratory epithelial cells (29). Pizzutto et al. measured a panel of pro-inflammatory cytokines, antimicrobial proteins, and cellular and clinical factors associated with airway inflammation in 70 children with CSLD (30). IFN-gamma was measured in PBMCs challenged *in vitro* with live NTHi. On multivariate regression, NTHi-specific IFN-gamma production was negatively associated with the BAL concentrations of IL-6 and IL-1beta. Therefore, in these patients, increased local airway inflammation was associated with impaired cell-mediated immune response to NTHi (30). In addition, in a study of adults with bronchiectasis, cytotoxic T cell demonstrated less IFN-gamma production *in vitro* to NTHi compared to controls (29). Collectively, this evidence suggests that an impaired cell-mediated immune response may lead to increased susceptibility to NTHi and contribute to the pathogenesis of CSLD, the precursor of bronchiectasis.

The immunologic milieu involved in the development of bronchiectasis specifically among patients with antibody deficiencies has not been well studied. It is possible that the previously described inflammatory processes in the lung are amplified, at least in some patients who have antibody deficiencies associated with immune dysregulation, such as CVID. In patients with CVID, bronchiectasis has been known to progress in the absence of obvious infections (31). The propensity toward autoimmunity and chronic inflammation in CVID is a proposed factor (32, 33). Insights which link the concepts of autoimmunity, chronic inflammation, susceptibility to lung infections, and the importance of neutrophils may suggest another clue regarding the pathogenesis of bronchiectasis (34). Specifically, the endotoxin-binding bactericidal/permeability-increasing protein (BPI) is present in leukocyte granules (35). It has antibacterial and anti-endotoxin properties (35). The presence of anti-BPI autoantibodies is associated with persistence of *Pseudomonas aeruginosa* and worse lung function in children with CF (34). In addition, anti-BPI auto-reactivity has been found to be strongly associated with the presence of anti-*P. aeruginosa* antibodies in two bronchiectasis cohorts in North America, suggesting that the breaking of tolerance to BPI is mediated through an association with *P. aeruginosa* infection (36).

Specific clinical and laboratory features have been found to correlate with the development of bronchiectasis in patients with antibody deficiencies as described in the “Identifying Bronchiectasis” section of this article. While such information may be helpful in knowing when to suspect bronchiectasis in a patient with an antibody deficiency, it sheds little insight into the underlying cause. Currently, our understanding of pathogenesis is limited mostly to extrapolation of studies examining the immune response in patients with bronchiectasis in general. As demonstrated above, there is a need for additional studies in patients with antibody deficiencies such as CVID, who have a predisposition to develop autoantibodies, yet cannot form protective antibodies appropriately. Understanding how underlying systemic immune dysregulation contributes to the development of bronchiectasis in patients with antibody deficiencies holds important potential implications for both prognosis and treatment.

## Identifying Bronchiectasis in Patients With Primary Antibody Deficiencies

Clinically, bronchiectasis presents with persistent or recurrent productive cough, airway infections, and pulmonary exacerbations (37). Although young children often do not expectorate, a wet-sounding cough is typically present and can be helpful in identifying patients suspected of having bronchiectasis (37). Children with recurrent episodes of protracted bacterial bronchitis (PBB), defined as a wet cough lasting at least 4 weeks which responds to antibiotics, are at risk for bronchiectasis, especially if they experience >3 episodes per year (38, 39). Lower airway infection with *H. influenzae* in patients with PBB was also found to be associated with development of bronchiectasis (39). Also, a wet or productive cough which fails to respond to 4 weeks of oral antibiotics predicts the presence of bronchiectasis (38). Patients with these atypical cough patterns should undergo evaluation for bronchiectasis, including consideration of underlying etiologies including antibody deficiencies (Table 1).

**Table 1:** Factors associated with Bronchiectasis in patients with antibody deficiencies.

Delayed diagnosis of antibody deficiency
History of pneumonia
Prolonged respiratory infections
Chronic wet or productive cough
Protracted bacterial bronchitis, >3 episodes per year
Abnormal or worsening pulmonary function testing
Difficulty maintaining sufficient IgG trough on IgRT
CD4 count <700 cells/microliter
Low B cells and/or Memory B cells
In the setting of CVID: very low IgA (<7 mg/dL) or very low IgM

**Figure 3:**
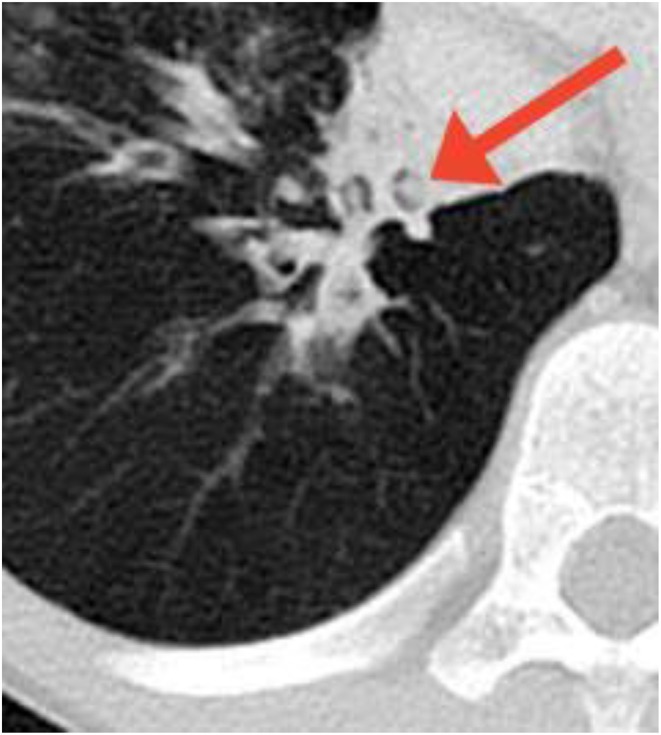
Bronchiectasis in a child with a previously undiagnosed antibody deficiency. Multiple dilated bronchi are evident on CT. Arrow denotes the signet ring appearance of an ectactic bronchus containing a mucous plug and adjacent artery with an abnormal bronchoarterial ratio > 1. Image courtesy David A. Manning, MD, Radiologist, Children's Hospital New Orleans.

**Figure 4:**
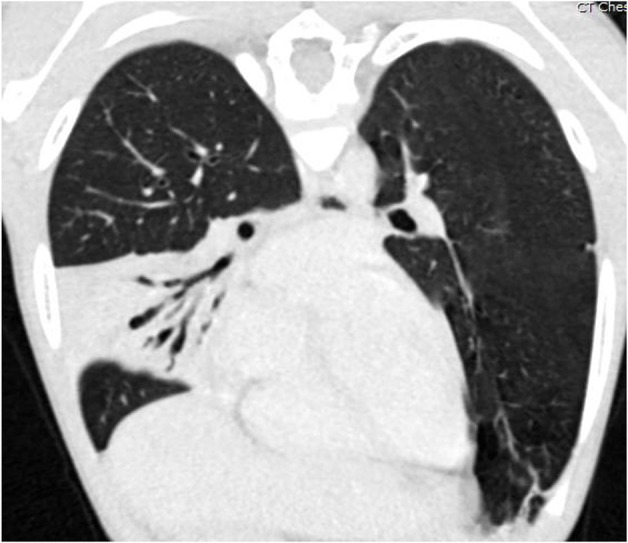
Coronal oblique reformatted CT image of the chest of the same patient as Figure 3, demonstrating air bronchograms within a right middle lobe consolidation with varicose bronchiectasis. Image courtesy David A. Manning, MD, Radiologist, Children's Hospital New Orleans.

Radiographically, high-resolution computed tomography (HRCT) is the most sensitive method for identifying structural lung abnormalities such as bronchiectasis. The predominant radiological feature of bronchiectasis is at least one dilated bronchus, defined as the internal luminal diameter of the airway exceeding the diameter of the adjacent vessel (9) (Figure 3). Additional key features include non-tapering of the bronchi as they traverse distally (Figure 4), and presence of visible bronchi within the outer 1–2 cm of the lung fields (9). While bronchiectasis is defined as a bronchoarterial ratio > 1, the normal anatomic ratio in children is much lower which may cause the diagnosis of bronchiectasis to be delayed or missed in this population (40). Some studies have suggested that the normal cut-off should be a ratio of 0.4–0.5 in infants and 0.8 in children less than age 18 years (9).

Among patients with antibody deficiencies, the decision of when to obtain the first HRCT and how often to repeat HRCT is a challenging exercise in clinical decision-making. In addition to the aforementioned clinical symptoms, other clinical characteristics and some immunologic abnormalities may be helpful in selecting which patients should receive a HRCT at the time their immunodeficiency is diagnosed. In one study, clinical factors which demonstrated a statistically significant correlation with the total CT score included length of respiratory symptoms before diagnosis of antibody deficiency, failure of immunoglobulin replacement therapy (IgRT) to establish adequate serum IgG levels, and FEV_1_ and forced vital capacity (FVC) of <80% predicted (41). Other associated factors include older age or diagnostic delay (7, 32) and prior history of pneumonia (42). Immune evaluation has demonstrated a correlation of bronchiectasis in the setting of CVID with a CD4 count <700 cells/microliter in peripheral blood (42). Other alterations in the lymphocyte subpopulations such as low IgM-memory B cells, class-switched-memory B cells, or total B cell number, also correlate with bronchiectasis (43). In patients with CVID, lower IgA (<7 mg/dL) (32) and lower IgM (mean 18 mg/dL with bronchiectasis vs. 26 mg/dL without bronchiectasis) (7) were associated with bronchiectasis (Table 1).

When deciding how often to repeat HRCTs, the clinician must consider that radiosensitivity and an increased risk for malignancy has been demonstrated in many forms of primary immunodeficiencies, including antibody deficiencies such as CVID (44–46). The cumulative dose of radiation due to diagnostic imaging that may be obtained in patients with a chronic condition could be excessive over time. A logical approach is to obtain HRCT no more often than 3–4 year intervals, unless an additional CT is needed to guide therapy or to evaluate new or worsening persistent symptoms (47). If a patient is maintaining a robust IgG trough level while on IgRT and is without chest infections, persistent cough, or decline in pulmonary function testing, it is appropriate to defer additional HRCT until a change in one of these factors is observed. Complete lung function testing including carbon monoxide diffusion at frequent intervals is a useful tool for monitoring the overall respiratory status in combination with clinical symptoms (47). Although pulmonary function testing is not sensitive enough to diagnose bronchiectasis, it can be useful for ongoing monitoring following the initial workup (47, 48). Any amount of ionizing radiation should be avoided unless it is absolutely essential to the care of the patient (49). It is important for the immunologist to lead the charge on this unique factor in the care of such patients, and to communicate the importance of radiosensitivity to the patient, pulmonologist, primary care provider, radiologist, and other team members.

To avoid exposure to ionizing radiation, magnetic resonance imaging (MRI) has been used among patients with exquisite sensitivity to radiation such as those with Ataxia-Telangiectasia, one of the many disorders involving DNA fragility or altered DNA repair mechanisms (37, 45, 49, 50). In addition to these patients, MRI has been shown to be comparable in detecting high and moderate grades of bronchial pathology among patients with CVID and may be considered as an alternative to HRCT (49, 51). Thoracic imaging with MRI has historically been underused due to technical barriers in obtaining high-quality images of the lungs but new techniques have been developed to overcome these challenges (52, 53). It is important to note that due to loss of signal in peripheral areas of the lung parenchyma, MRI is less sensitive than HRCT in detecting peripheral bronchial alterations (51). In addition, bronchiectasis may be more difficult to detect on MRI if not associated with a thickened wall or centered within an area of lung consolidation (53). While HRCT remains the most sensitive modality in detecting bronchiectasis (51, 54, 55), MRI can play an important role in monitoring patients with moderate or severe structural abnormalities (49, 52). In addition, MRI may be more sensitive in detecting early airway wall changes and mucus retention that may precede more serious structural changes such as bronchiectasis (56).

## Optimizing Immunoglobulin Replacement Therapy

It has been demonstrated that patients who continue to develop respiratory infections are at increased risk for progression of bronchiectasis (43). For patients who have an inability to make a strong antibody response, IgRT is the most important tool to protect against chest infections and subsequently slow this progression. Higher IgG trough levels, in addition to protecting against pneumonia (57), may also have a protective benefit against silent progression of bronchiectasis (31). Trough levels should never be allowed to fall below the physiologic normal for age. In fact, a trough of 1,000 mg/dL may be considered optimal for patients with bronchiectasis (31, 57). In children, a trough of 800 mg/dL may be sufficient, as long as they are not experiencing recurrent chest infections with their current IgRT plan. In addition to increasing the dose, techniques such as shortening the interval between infusions or changing to subcutaneous IgG (SCIg) replacement may be useful in achieving appropriate trough levels. Despite these recommendations for therapeutic trough levels, it is important to note that there is a wide range of IgRT doses which have been shown to keep patients with antibody deficiencies infection-free (58). In addition, patients with bronchiectasis may require twice as much replacement to achieve the same trough level as those without, possibly secondary to either increased losses or metabolism of IgG (58). A practical starting point would be no less than 0.6 g/kg/month and if the patient develops 3 or more infections a year, the dose should be increased (58). IgRT should thus be individualized and adjusted as necessary with a goal of preventing breakthrough infections rather than a sole focus on target trough levels (58). Despite treatment with immunoglobulin, patients with agammaglobulinemia and CVID may still develop chronic lung disease (59) and may need prolonged or prophylactic antibiotics in order to place a halt on the recurrent lung infections. It is essential for both the care team and the patient to understand that while IgRT is exceedingly important, an effective treatment plan for bronchiectasis is multifaceted. An aggressive multidisciplinary approach, discussed throughout this article, is also essential.

## A Multifaceted Approach to Pulmonary Management

### General Principles

**Figure 5:**
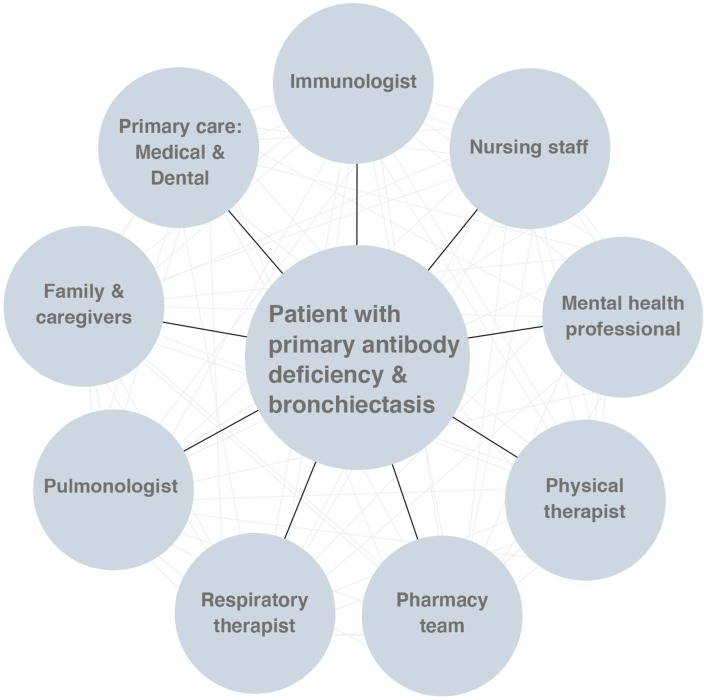
The multidisciplinary approach to the care of patients with primary antibody deficiency and bronchiectasis.

The central intent of the management of bronchiectasis is to minimize ongoing damage to the airways, and this is especially critical for pediatric patients, in whom the lungs are still developing (38, 60). With appropriately-intensive management and monitoring from subspecialist providers, and a commitment to self-care regimens from patients and families, the progression of bronchiectasis can be slowed, and the worst outcomes potentially avoided. It is important for each member of the multidisciplinary team to have at least a basic concept of the overall scope of the patient care plan. Clear communication with the patient and between team members is essential (Figure 5).

Bronchiectasis manifests over a spectrum of clinical severity, and pulmonary management is best accomplished when tailored to the individual patient. Current usual management may include the long-term use of inhaled pro-mucogenic and antibiotic therapies, inhaled bronchodilators, chronic anti-inflammatory use, physiotherapeutic airway clearance techniques, pulmonary rehabilitation services, and, in those cases where there is marked structural damage, surgery (61). Optimal treatment consists of restoring or approximating normal airway clearance and facilitating mucociliary escalator function; and many patients will require acute interventions for intermittent bronchiectasis exacerbations. A typical approach to the use of multiple combined therapies is to use a short-acting bronchodilator to open the airways, followed by an inhaled muco-active hyperosmotic agent, then a mechanical airway clearance technique. Finally, after the airway has been optimized, inhaled medications (e.g., antibiotics) are used when appropriate (61). The best airway clearance regimen for the patient is not merely the one which is found clinically to be most effective, but also the one to which a given patient can best adhere. As such, the development of a patient's home regimen is a collaborative and longitudinal project between the patient and caregivers.

While the evidence base for the management of non-CF bronchiectasis is growing, there is a paucity of robust research regarding many of the techniques described below, particularly in the specific population of primary antibody deficiency (62). While many of these therapeutic approaches have well-established benefit in the CF population, this may or may not be portable to patients with non-CF bronchiectasis. A notable example of once such disjunction was the attempted use of recombinant DNAse in the non-CF bronchiectasis population, which was demonstrated in a clinical trial to actually increase the frequency of pulmonary exacerbations in these patients (63). To date, systematic reviews on the management of non-CF bronchiectasis have yielded only conditional recommendations based on relatively low-quality evidence. In light of the lack of strong randomized-controlled trials of bronchiectasis therapies, much of the current standard of care for this disease is based on expert opinion and consensus. The European Respiratory Society (ERS) and British Thoracic Society have published recent guidelines on the management of bronchiectasis (61, 64). These important consensus statements form the basis for the subsequent discussion in this section. There are presently no consensus guidelines for the management of bronchiectasis in patients with primary antibody deficiency (62).

### Mechanical Airway Clearance Therapies

The inspissated mucus of the bronchiectatic airway is a risk factor for bacterial colonization in the lung and contributes to the “vicious cycle” of infection and inflammation in patients (65). As mucociliary clearance is often dramatically reduced in non-CF bronchiectasis, several airway clearance techniques (ACTs), both provider- and self-administered, are deployed to help patients mobilize and expectorate mucus. While any of these techniques may be clinically and subjectively helpful to a given patient, it is important to note that most are not supported by robust evidence (66). Current expert consensus recommends that patients with chronic cough and difficulty expectorating be taught a preferred airway clearance technique, which they should perform twice daily as part of a broader airway clearance regimen (61, 67). There is currently no guidance as to which technique is preferred, and so a certain amount of pragmatism should be maintained: The best airway clearance technique is the one which works for the patient and to which they will adhere (67). Review of CT imaging may complement any manual physiotherapy regimen by focusing attention and time on the affected lobes (64).

#### High-Frequency Chest Wall Oscillation Therapy

High-frequency chest wall oscillation (HFCWO) therapy, performed either by a respiratory therapist with percussive panels connected to an air-pulse generator or by wearing a close-fitting inflatable vest similarly connected to an air-pulse generator, works by breaking up and thinning mucus in the airways through the oscillations of the chest wall and lung parenchyma. Though these devices have been studied extensively in the CF population, evidence is mixed and cannot be easily extrapolated to the non-CF bronchiectasis population (68).

#### Manual Chest Physiotherapy

Manual chest physiotherapy is a specialized airway clearance technique wherein a physical therapist or trained caregiver rapidly percusses the chest wall with cupped hands. This manual percussion and movement of the chest wall is usually combined with assisted postural drainage to optimize the proximal movement and subsequent expectoration of mucus. Systematic review and meta-analysis of this technique in CF patients demonstrated no advantage of manual chest percussion physiotherapy over other airway clearance techniques, including self-administered therapies (69). Nevertheless, when a patient is admitted to the inpatient service, a physical therapy consultation could be considered for assistance in airway clearance.

#### Expiratory Flow Modification and Autogenic Drainage

In addition to manual chest physiotherapy administered by a caregiver or therapist, patients may learn self-administered airway clearance techniques including specialized forced-expiration breathing patterns and self-directed postural drainage. These techniques are particularly helpful in that they are imminently portable (i.e., requiring no equipment) and the associated feelings of self-efficacy can be empowering to patients. These methods are comparable in clinical efficacy (including measures of pulmonary function and sputum production) to other airway clearance techniques, and may be preferred by patients (70).

#### Positive Expiratory Pressure Devices

Positive expiratory pressure devices work by providing slight resistance against exhalation, which further opens mucus-impacted airways through gentle additional positive end-expiratory pressure (PEEP). Some patients prefer to use handheld vibratory positive expiratory pressure devices, which similarly provide distending PEEP while adding the element of vibratory airflow which is thought to help break up mucus and move it up the mucociliary ladder. PEEP devices have compared favorably to HFCWO therapy in systematic review, particularly in terms of reduction in pulmonary exacerbation frequency and in patient satisfaction (71).

#### Bronchoscopy

Though not a part of routine bronchiectasis management, select patients may benefit from early bronchoscopy with lavage. When clinically warranted, flexible bronchoscopy with therapeutic lavage serves the dual role of permitting some mechanical clearance of mucus in the larger airways while generating potentially useful bronchial lavage samples for cellular and microbiologic analysis. Close communication with the attending anesthesiologist to promote lung recruitment during sedation and recovery is necessary.

### Exercise and Pulmonary Rehabilitation

Maintenance of daily physical activity, optimally including some element of aerobic exercise, is an important part of bronchiectasis management. Current consensus recommendation is that adult bronchiectasis patients with impaired exercise capacity should undergo a pulmonary rehabilitation program in addition to daily airway clearance therapies (61). Indeed, this single recommendation from the 2017 ERS consensus statement was the only deemed to be supported by high-quality evidence (61, 64). When hospitalized, it may be helpful to consult physical therapy for exercise, if they are not already involved in the case for airway clearance.

### Inhaled Therapies

Inhaled therapies, both as nebulized solutions and small-particle inhalers, are an attractive mode of medication delivery, as they offer deposition of medication directly to the airways. In the management of bronchiectasis, this is potentially complicated by the heterogeneity of ventilation and mucus in the airways; nevertheless, inhaled medications are a central component to most management regimens.

The use of these medications, which are generally scheduled twice daily to co-occur with a mechanical airway clearance therapy, is time-consuming and difficult to perform outside of the home. As a consequence, inhaled medication regimens should be tailored to the individual patient, with consideration for the patient's lifestyle, preferences, and severity of disease. Current expert consensus suggests testing patient tolerance to a given inhaled agent before committing to a course of therapy (61). Broadly, the most common adverse effects of these medications are throat irritation and bronchoconstriction, and premedication with a beta-agonist is reasonable in select cases (61).

#### Hyperosmolar Agents and Mucolytics

The use of inhaled mucolytic medications and hyperosmolar solutions is central to bronchiectasis care in the management of CF, however the current evidence base is mixed in non-CF bronchiectasis (72). Nevertheless, expert consensus favors the use of nebulized pro-mucogenic agents in those non-CF bronchiectasis patients with difficulty adequately mobilizing secretions (61, 64). Of note, by design, these mediations aid with the mobilization and expectoration of mucus, and occasionally patients perceive the resultant increase in mucus production as aversive.

Nebulized saline, generally as a hypertonic solution (>0.9% w/v, and usually as 3 or 7% in clinical practice), hydrates airway mucus by osmotic action, pulling water from the airway epithelium, improving mucus viscosity, and enhancing mucociliary clearance. Patients generally perform nebulizer treatments twice daily concurrent with their preferred airway clearance technique, with each treatment taking up to 30 min. One randomized single blind cross-over study of 28 adult patients with non-CF bronchiectasis found a clinically meaningful and statistically significant improvement in FEV_1_, FVC, and a QOL score, and reduced antibiotic usage and emergency health care utilization (73). In patients with milder disease, isotonic saline, or even sterile water may be equally effective and may be better tolerated in those patients who experience bronchospasm with hypertonic saline (64, 72).

Recombinant DNAse (dornase alfa), works principally by lysing extracellular DNA aggregates resulting from the NETosis of neutrophils, which makes it attractive for use in disorders which involve a dysregulated neutrophil response, such as bronchiectasis. Unfortunately, while DNAse is an impactful therapy for many CF patients, it was associated with an increased frequency of exacerbations and worsened FEV_1_ in a clinical trial in idiopathic bronchiectasis patients (63). As such, its use in the adult non-CF bronchiectasis population is *explicitly discouraged* by current guidelines (61, 64).

#### Inhaled Bronchodilators

The use of short-acting inhaled bronchodilators prior to a patient's airway clearance regimen is reasonable, as this both opens the airways prior to subsequent inhaled therapies and mechanical airway clearance and may limit bronchoconstriction related to the use of inhaled hyperosmotic agents and antibiotics. The use of long-acting bronchodilators as a maintenance therapy should not be routinely offered to patients without another strong clinical indication (e.g., significant breathlessness, COPD) (61).

#### Inhaled Antibiotics

A national registry of non-CF bronchiectasis patients in the United States of America found that *P. aeruginosa, Staphyolococcus aureus, H. influenzae*, and non-tuberculous *mycobacteria* (NTM) are the commonest bacterial pathogens in this population (74). Long-term courses of inhaled antibiotics (e.g., colistin, tobramycin, and gentamycin) should be considered for patients with *P. aeruginosa* infection and 3 or more pulmonary exacerbations in a year, or for those patients without *P. aeruginosa* who have frequent exacerbations which are macrolide-refractory, or for those patients who are intolerant of oral macrolides (61). There is no strong evidence favoring oral vs. inhaled antibiotics in bronchiectasis, and so clinical judgement must be used in concert with attention to patient preferences (75). Possible adverse effects from inhaled antibiotics used in bronchiectasis include throat irritation and dysgeusia, chest discomfort and bronchoconstriction, and cough. Serum levels are generally not required for monitoring of therapeutic index when using inhaled antibiotics (i.e., aminoglycosides) alone in patients without underlying renal disorders, but should be considered if there is clinical evidence of nephrotoxicity or ototoxicity (e.g., tinnitus) (76, 77).

#### Inhaled Corticosteroids

As inflammation is central to the pathomechanism of bronchiectasis, it seems reasonable to include an inhaled corticosteroid (ICS) in the management of these patients. To date, relatively small trials of ICSs in bronchiectasis patients have not demonstrated benefit in either reducing the frequency of exacerbations or in improving pulmonary function or QOL scores. Nevertheless, ICS use is rather common in adult patients with bronchiectasis (78). A Cochrane review of seven studies involving 380 adult patients concluded that there was insufficient evidence to support use of ICS in adult bronchiectasis patients (79). This conclusion is consistent with current consensus expert guidelines, which state that ICS should not be routinely offered to adults with bronchiectasis, but that patients with comorbid asthma or chronic obstructive pulmonary disease (COPD) should continue these medications if deemed clinically useful (61, 64).

### Systemic Therapies

#### Systemic Antibiotics for Acute Exacerbations

Systemic antibiotics, generally taken orally, are a mainstay in the management of acute exacerbations of bronchiectasis. Optimally, the antibiotic management of bronchiectasis is informed by the patient's own usual pulmonary pathogens and chronic colonizers. When clinically feasible, a sputum sample should be obtained prior to initiation of antibiotic therapy to surveil for changes in susceptibility patterns. Additionally, a functional understanding of any underlying immune defect may inform antibiotic choice and duration of therapy. However, in clinical practice empiric antibiosis is often pursued. In this case, macrolides (e.g., azithromycin, erythromycin) are considered first-line and have been shown to significantly reduce the frequency of pulmonary exacerbations (61, 80). Current expert consensus recommends 14-day courses of systemic antibiotics for bronchiectasis exacerbations in adults, citing no direct evidence of benefit from longer courses, and the need to balance patient preference and the risk of inducing antibiotic tolerance (61). Systematic review of controlled trials examining the utility of concurrent use of inhaled and oral antibiotics for exacerbations found no evidence of benefit for this wide-spread practice (81).

While induced sputum samples may yield helpful cultures in older patients, in younger patients sputum cultures may over-represent oropharyngeal flora. Flexible bronchoscopy with thorough lavage can yield directive bacterial culture and antibiotic susceptibility panels, though it should be remembered that there is potentially marked heterogeneity in bacterial sub-populations within different segments of the airways and lungs.

Eradication antibiotic protocols for those patients with newly-identified *P. aeruginosa* should be pursued (61). This recommendation is due to the markedly deleterious effect of *P. aeruginosa* on the rapidity of decline in pulmonary function in bronchiectasis patients. *P. aeruginosa* eradication regimens generally involve a two-week initial treatment phase with oral or intravenous antibiotics and an adjunctive inhaled antibiotic (e.g., colistin, tobramycin, or gentamicin), followed by an additional 10 weeks of inhaled antibiotics. Such a regimen is not currently recommended for other pathogens associated with bronchiectasis.

#### Systemic Antibiotics for Prophylaxis

As discussed previously, despite appropriate immunoglobulin replacement, many patients with antibody deficiencies will still develop bronchiectasis. Patients who should be considered for prophylactic antibiotics include those with three pulmonary infectious exacerbations in 1 year (61, 82, 83) or who have declining lung function despite appropriate immunoglobulin replacement (84, 85). Because macrolide antibiotics have anti-inflammatory and anti-microbial properties, they are most commonly used for prophylaxis in patients with bronchiectasis (86–88). Typical dosing includes 5 mg/kg for children or 250 mg/day for adults three times per week (2, 89). The use of prophylactic antibiotics in patients with primary immunodeficiency and bronchiectasis is primarily extrapolated from CF and non-CF bronchiectasis, as there is little published data specifically on patients with antibody deficiencies. A Cochrane review in 2015 evaluated the evidence behind courses of prolonged (≥1 month) antibiotic therapy for non-CF bronchiectasis (90). This meta-analysis of 18 randomized controlled trials and 1,157 patients (including mainly adults and a smaller subset of children) suggested that prolonged antibiotic use was associated with a significant reduction in the number of reported bronchiectasis exacerbations, and a non-statistically significant reduction in hospitalization. Overall, the authors found that there is evidence of moderate quality to suggest that the use of prolonged antibiotics may benefit patients with non-CF bronchiectasis, however they did raise concerns about the potential for emergence of antibiotic tolerance with this approach (90). Subsequent systematic reviews and meta-analyses reaffirmed prior findings of a reduction in exacerbation and improvement in QOL scores and pulmonary function indices (82, 91). Two large recent randomized control trials, the EMBRACE (Azithromycin for prevention of exacerbations in non-CF bronchiectasis) and BAT (Bronchiectasis and Longterm Azithromycin Treatment) trials, also endorsed the role of azithromycin in non-CF bronchiectasis, the latter of which included five patients with CVID (92, 93). Due to the risks of antimicrobial resistance, treatment should be discontinued after a trial of 3–6 months if there is no clear evidence of benefit (83). Prior to initiating long-term macrolides, it is important to rule out non-tuberculous mycobacterium (64).

#### Immune-Modulating and Anti-inflammatory Medications

Vaccination is an important consideration in the preventative care of children and adults with bronchiectasis. Expert opinion recommends giving the seasonal *influenza* vaccination, as well as ensuring maintenance of vaccination against *S. pneumonia, H. influenzae*, and *Bordetella pertussis* when appropriate for the patient and not otherwise contraindicated (94–97). Most patients with antibody deficiencies are maintained on IgRT and are receiving antibody protection passively. Therefore, the above-mentioned vaccines are not indicated while on an IgRT plan, apart from influenza vaccination. Because influenza virus antigens change frequently and there is a significant delay between the time of donor IgG collection to the production of commercially available IgRT, IgRT might not contain the most seasonally relevant anti-influenza antibodies (98).

### Surgical Management

In some patients, bronchiectasis may develop in sub-segmental airways to an extent where distal lung segments are no longer functional. These areas may then act as a reservoir for chronic infection, the development of highly resistant pathogens, and episodic reinfection of adjacent lung units. When such disease is highly localized, and when optimal medical management has failed to improve a patient's pulmonary health, surgical resection of the affected lobe (or, less commonly, segmentectomy or pneumonectomy) can be considered. Advances in the medical management of bronchiectasis have made surgical intervention less common. However, in the case of highly-localized disease, resection of affected lobes can improve a patient's QOL and reduce the frequency of antibiotic courses (99, 100). Indication for surgical resection of an affected lobe of the lung include persistent failure of medical management and hemoptysis, and current expert consensus recommends limiting lobectomy to those patients in whom maximal medical management has been trialed (61, 100).

Pre-operative management should include 1–2 weeks of targeted antibiosis to avoid complications including bronchopleural fistula and empyema (101). Generally, performance of contrast-enhanced CT to reassess the extent of disease and aid surgical planning is warranted (101). It is important to note that, following any surgical intervention to the upper airway, chest, or abdomen, pain may limit effective airway clearance through cough, and the use of opioid pain medications could suppress the respiratory drive, particularly during sleep. In these cases, a physical therapy consultation can assist with adaptive airway clearance techniques (e.g., cough splint) during recovery to minimize atelectasis.

One consideration for those patients in whom aspiration is felt to be a driver or major contributor to the treatment-refractoriness of their bronchiectasis, is correction of anatomical issues promoting reflux and aspiration, including fundoplication and laryngeal cleft repair (102–104). Aspiration may cause both direct tissue damage and bacterial inoculation of the airways, but could also modulate the growth and lifestyle patterns of pathogens in the airways, driving them to more virulent or chronic expression patterns (105). Prokinetic medications and conservative anti-reflux precautions should be considered first (64).

Lung transplantation referral may be considered for those patients 65 years old or less with FEV_1_ <30% predicted, or in those patients with rapid functional decline and severe pulmonary hypertension (pHTN), massive hemoptysis, or respiratory failure (64). Lung transplantation has been used in patients with CVID with variable results (28, 47, 106).

### Management of Extra-Pulmonary Complications of Bronchiectasis

Patients with bronchiectasis may have significant comorbid extra-pulmonary health issues, including failure to thrive and psychosocial problems. Primary antibody deficiency patients with bronchiectasis are at increased risk for pHTN, and so echocardiography should be considered in those patients with dyspnea, exercise intolerance, and other signs of pHTN (107). Sinus disease is common in this population and should be managed when found as the sinus can act as a reservoir for sinopulmonary pathogens. Likewise, good dental hygiene and routine dental visits are important to reduce oral infections.

### Longitudinal Follow-Up and Patient Adherence to Chronic Therapy

Patients with bronchiectasis generally warrant close follow-up, as daily airway clearance regimens are, by their nature, time-consuming, and often difficult to maintain. Clinic visits should include an honest, direct reappraisal of how the patient and family is *actually* performing their regimen, and the clinician should be prepared to troubleshoot when things are not working well or are impracticable. Close follow-up in clinic also permits the monitoring of pulmonary function, and a decline in FEV_1_ and related indices may herald an impending exacerbation and provide an opportunity for early, outpatient intervention. While there is no specific consensus guidance for primary antibody deficiency patients with bronchiectasis, other subspecialty organizations recommend clinic visits as frequently as four times annually, and this may be appropriate for primary antibody deficiency patients with significant bronchiectasis (108). These visits should include repeat pulmonary function testing, sputum microbiology, and consideration of chest x-ray (64).

In one study of a population of 75 patients with non-CF bronchiectasis with a sputum culture positive for *P. aeruginosa*, researchers found that beliefs about the medical necessity and potential risks of medications and airway clearance techniques predicted adherence to therapy (109). Older age was associated with increased adherence, as was fewer medications in the regimen. A structured literature review published by the Cochrane group in 2015 found no studies which tested interventions to improve adherence, and we were not able to identify any subsequent clinical studies on this topic (110).

## Future Directions in Bronchiectasis Management

Though much of the existing evidential support for therapies in non-CF bronchiectasis is relatively weak, the field of research has grown appreciably in the past few years, and several promising avenues of treatments have been identified. A recent review by Chalmers and Chotirmall has capably mapped the current state of the field by identifying 11 clinical trials comprising the cutting-edge of current research (111). Among these, perhaps the most mechanistically compelling are those targeting immune dysregulation and off-target effects in the bronchiectatic airway, such as the current trial of a human NE inhibitor (BAY 85-8501) (25). Doubtless, the evidentiary basis for our interventions will continue to strengthen in the coming years as these clinical trials progress. New patient registries will be invaluable in drawing new insights into this previously under-studied disease (112).

To best guide management and anticipate bronchiectasis exacerbations, new diagnostic and monitoring techniques will need to be developed. The addition of molecular techniques to sputum microbiology assays offers improved sensitivity in pathogen detection, and a window into the complex pulmonary microbiome (113). One study of sputum NE activity in a large population of adults with bronchiectasis found an association of increased activity and future risk of exacerbation, offering the promise of an easily-obtainable bio-marker for clinical surveillance (24, 114). There is robust research presently underway to identify clinical phenotypes and so-called “treatable traits” in non-CF bronchiectasis, which will one day permit more precise and tailored management strategies (115, 116).

As it is likely that daily maintenance interventions for bronchiectasis will remain relatively involved for patients and caregivers despite these ongoing advancements, it will be important to study ways to foster adherence in this population (110). Chronic bronchiectasis and primary antibody deficiency are burdensome diseases for patients and their families, and research into psychosocial interventions for these patients is needed. Expansion of multi-disciplinary care for patients with primary antibody deficiency-associated bronchiectasis (i.e., building teams of immunologists, pulmonologists, nurses, mental health professionals, case-workers, and respiratory and physical therapists), is critical to ensuring that health outcomes for these patients meet or exceed those for patients with bronchiectasis secondary to other etiologies (117–119) (Figure 5).

## Author Contributions

LW designed the concept of the manuscript, contributed to the writing, and editing of the manuscript. EW contributed to the writing and editing of the manuscript. KG contributed to the writing and editing of the manuscript, and the design of figures. RS provided oversight and critical review of the manuscript.

### Conflict of Interest

The authors declare that the research was conducted in the absence of any commercial or financial relationships that could be construed as a potential conflict of interest.
